# PD1 blockade enhances cytotoxicity of *in vitro* expanded natural killer cells towards myeloma cells

**DOI:** 10.18632/oncotarget.10235

**Published:** 2016-06-23

**Authors:** Yanan Guo, Xiaoli Feng, Yang Jiang, Xiaoyun Shi, Xiangling Xing, Xiaoli Liu, Nailin Li, Bengt Fadeel, Chengyun Zheng

**Affiliations:** ^1^ Hematology Department, The Second Hospital of Shandong University, Jinan, China; ^2^ Institute of Biotherapy for Hematological Malignancies, Shandong University, Jinan, China; ^3^ Shandong University-Karolinska Institutet Collaborative Laboratory for Stem Cell Research, The Second Hospital of Shandong University, Jinan, China; ^4^ Clinical Laboratory Department of The Second Hospital, Shandong University, Jinan, China; ^5^ Department of Medicine-Solna, Clinical Pharmacology Group, Karolinska Institutet, Karolinska University Hospital-Solna, Stockholm, Sweden; ^6^ Karolinska Institutet, Institute of Environmental Medicine, Division of Molecular Toxicology, Stockholm, Sweden; ^7^ Childhood Cancer Research Unit, Department of Women's and Children's Health, Karolinska Institute, Karolinska University Hospital, Stockholm, Sweden

**Keywords:** natural killer cells, in vitro expansion, PD1, anti-tumor activity, myeloma

## Abstract

Aiming for an adoptive natural killer (NK) cell therapy, we have developed a novel protocol to expand NK cells from peripheral blood. With this protocol using anti-human CD16 antibody and interleukin (IL)-2, NK (CD3^−^CD56^+^) cells could be expanded about 4000-fold with over 70% purity during a 21-day culture. The expanded NK (exNK) cells were shown to be highly cytotoxic to multiple myeloma (MM) cells (RPMI8226) at low NK-target cell ratios. Furthermore, NK cells expanded in the presence of a blocking antibody (exNK+PD1-blockage) against programmed cell death protein-1 (PD1), a key counteracting molecule for NK and T cell activity, demonstrated more potent cytolytic activity against the RPMI8226 than the exNK cells without PD1 blocking. In parallel, the exNK cells showed significantly higher expression of NK activation receptors NKG2D, NKp44 and NKp30. In a murine model of MM, transfusion of exNK cells, exNK+PD1-blockage, and exNK plus intratumor injection of anti-PD-L2 antibody (exNK+PD-L2 blockage) all significantly suppressed tumor growth and prolonged survival of the myeloma mice. Importantly, exNK+PD1-blockage presented more efficient therapeutic effects. Our results suggest that the NK cell expansion protocol with PD1 blockade presented in this study has considerable potential for the clinical application of allo- and auto-NK cell-based therapies against malignancies.

## INTRODUCTION

Natural killer (NK) cells, a group of large granular lymphocytes, play essential roles in immune system against malignant and infected targets [1]. Unlike T cells, NK cells act as innate immune cells, and conduct rapid and selective “natural” killing of cellular targets (tumor cells and infected cells), which lack major histocompatibility complex (MHC) class I expression, without prior sensitization steps. Interestingly, NK cell memory-like response to viruses has also been demonstrated by recent studies, indicating an adaptive immune traits of NK cells [2]. In view of their multiple roles in interaction with other immune components, NK cells are considered to be an important immune player in the fight against tumors and infected cells through exerting direct and indirect cytotoxicity, and shaping adaptive immune response [3].

Recognition of target cells is governed by a complex set of activating and inhibitory receptors expressed on NK cell surface. The balance between activation and inhibition signaling decides the outcome of NK cell cytolytic response to target cells [4]. Parallel to the improved understanding of NK cell biology, NK cell-based immune therapies in clinical settings have also been developed, improved, and tested in cancer patients, including hematological cancers, and some promising results have been reported [5]. NK cells for immunotherapies can be obtained from autologous peripheral blood mononuclear cells (PBMC) or allogeneic sources, such as umbilical cord blood (UCB), NK lymphoma cell lines, as well as other types of pluripotent stem cells [6, 7]. Owning to the “missing-self” characteristics of NK cell activation, allogeneic NK cell transfusion resulted in more promising outcome than autologous ones in the treatment of cancer patients [8]. As MHC class I molecules are ligands for most inhibitory receptors expressed on NK cells, engagement of MHC-I on target cells with inhibitory receptors delivers inactivation signaling to NK cells, resulting in survival of the target cells. In contrast, when there is a mismatch between an inhibitory subgroup of killer immunoglobulin-like receptors (KIRs) on NK cells and MHC-I on target cells, NK cells get activated and the target cells get killed [9]. In line with this hypothesis, KIR-ligand mismatched NK cells in haploidentical stem cell transplantation (SCT) and allo-NK cell transfusion settings exert significant anti-leukemia effects and/or survival improvement [10–14]. To a lesser extent, certain beneficial effects of autologous NK cell transfusion on hematological malignancies and solid tumors have also been reported [15–17].

NK cell only accounts for 5-15% of peripheral lymphocytes (PBL). Apparently, developing a protocol for efficient activation and expansion of NK cells to achieve sufficient number of functional NK cells is curial for adoptive allo- and auto-NK cell therapies. Various methods using antibodies and cytokines with or without feeder cells to expand NK cells ex vivo have been reported [18–24]. Induction of NK cell activation is the key step for NK cell expansion. CD16 is one type of Fcγ receptors, a low-affinity receptor (FcγRIIIa) for the Fc portion of immunoglobulin, and cross-linking of CD16 to target cells induces NK cell antibody-dependent cellular cytotoxicity (ADCC) and lysis of the target cells [25]. Binding of CD16 agonist anti-CD16 antibody on NK cells is capable of triggering NK cell activation that results in NK cell cytotoxic activity against cancer cells and releasing of cytokines, such as interferon γ (IFNγ) [25, 26]. Moreover, blocking CD16 cleavage by inhibiting ADAM metallopeptidase domain 17 (ADAM17) to boost NK cell cytotoxicity towards cancers has been suggested by a recent study [27]. Therefore, activation of NK through CD16 can be a promising strategy for *in vitro* induction of NK cell activation and expansion.

Targeting on immune checkpoint molecules such as programmed cell death protein 1 (PD1) and its ligands PD-L1 and PD-L2 by antibodies to block their inhibitory signaling has achieved great success in treatment of several solid tumors and hematological malignancies [28–33]. Engagement of PD1 with PD-L1/L2 expressed on antigen presenting cells (APCs) delivers inhibition signaling, and this negative regulation of immune response pathway plays crucial roles in induction and maintenance of peripheral immune tolerance [34]. In symptomatic cancer patients, T cells in tumor microenvironment often express PD1, and interaction between PD1 and PD-L1 on cancer cells creates a network blocking T cell-mediated eradication of cancer cells [35–38]. Such PD1 positive T cells are considered to be a group of exhausted T cells, characterized by reduced effector function and proliferation index [39]. In addition to the findings observed in T cells, NK cells from cancer patients such as multiple myeloma (MM) were also shown to express PD1 [40]. Concerning PD1 expression on T cells is inducible upon T cell priming, it is presumable that *in vitro* activation and expansion procedures may also induce and up-regulate PD1 expression on NK cells. Therefore, it would be of great interest to evaluate PD1 expression on NK cells and the functional changes of NK cells in relation to PD1 blockage in a NK cell expansion system.

MM is a hematologic tumor characterized by an uncontrolled clonal expansion of malignant plasma cells [41]. With the development and clinical application of new anti-MM drugs, such as bortezomib and lenalidomide, outcome of MM therapy has been markedly improved, but MM still remains incurable. Similar to other malignancies, relapse cannot be effectively prevented due to minimal residue disease (MRD), in which those remaining cancer cells are usually resistant to conventional therapies. Immunotherapies including NK cell transfusion in combination with PD1/PD-L1/2 blockage may offer a potential solution for eradication of MRD in MM and other tumors.

Here, we demonstrated that NK cells from PBMCs of healthy donors could be efficiently expanded using a protocol employing anti-CD16 antibody and interleukin (IL)-2, with an expansion of about 4000-fold and a purity of over 70% after a 21-day culture. More importantly, the effector function of *in vitro* expanded NK cells (exNK) was significantly enhanced, and their PD1 expression was also increased. Furthermore, adding anti-PD1 antibody to the expansion system substantially improved the exNK cell cytotoxic activity towards myeloma cell line RPMI8226. Consistent with the *in vitro* findings, exNK+PD1-blockage more efficiently controlled the myeloma tumor mass and prolonged survival of myeloma mice than other treatment remedies. These results suggest that incorporation of PD1 blockade to the NK cell expansion protocol may have considerable value in improving NK cell-based therapy for MM and other malignancies, and that the therapeutic effects of *in vitro* expanded NK with PD1 blockage deserve a clinical trial in MM and other malignancies.

## RESULTS

### NK cell expansion from PBMCs of healthy donors

**Figure 1:**
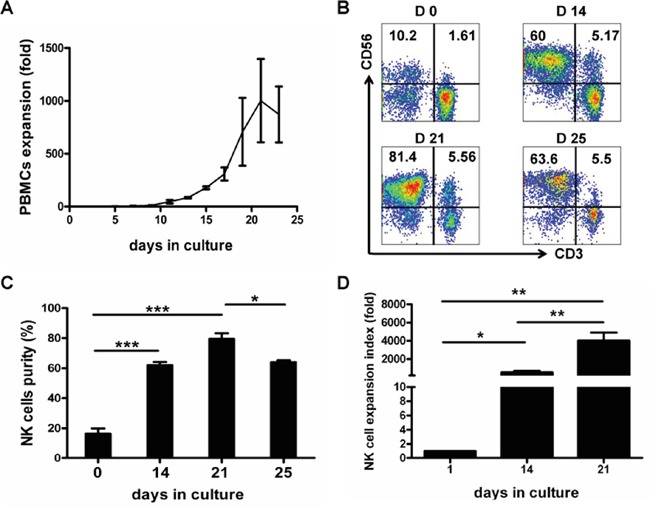
Time course of *in vitro* expansion of PBMCs and NK Cells Mononuclear cells from healthy blood donors (PBMCs) were collected and PBMCs were activated and expanded by using our defined protocol as described in the Materials and Methods. PBMCs and NK cell expansion fold and purity were analyzed at various culture time-points indicated. **A.** Time course of PBMCs expansion. Results of three independent experiments are presented as mean ± SEM. **B.** Dot plots from one representative experiment depicting NK cell (CD3^−^CD56^+^) purity. **C.** Results of NK cell purities are shown as mean ± SEM from 3 independent experiments (*P<0.05, ***P<0.001, by Student's *t* test). **D.** Results of NK cell expansion folds from 7 independent experiments are presented as mean ± SEM (*P<0.05, **P<0.01, by Student's *t* test).

Three independent experiments were first performed to determine the time course of an optimal expansion. As shown in Figure 1A, expansion rate of PBMCs peaked on day 21 of PBMC culture, with the cell number increased by 1002.2±394.53-fold. Flow cytometric NK cell phenotyping showed that NK cell purity (CD3^−^CD56^+^) also reached the peak (79.6%±3.7%) on day 21 of culture (Figure 1B and 1C). Furthermore results from seven independent experiments showed that NK cells were expanded by 549.9±154.7-fold on day 14 and by 4011.5±1082.4-fold on day 21, and that NK expansion rate on day 21 was significantly higher than that on day 14 (*P*<0.01) (Figure 1D). Therefore, expanded NK cells of 21-day-culture were used for all the following experiments.

### Surface expression of activating and inhibitory receptors on the NK cells

**Figure 2:**
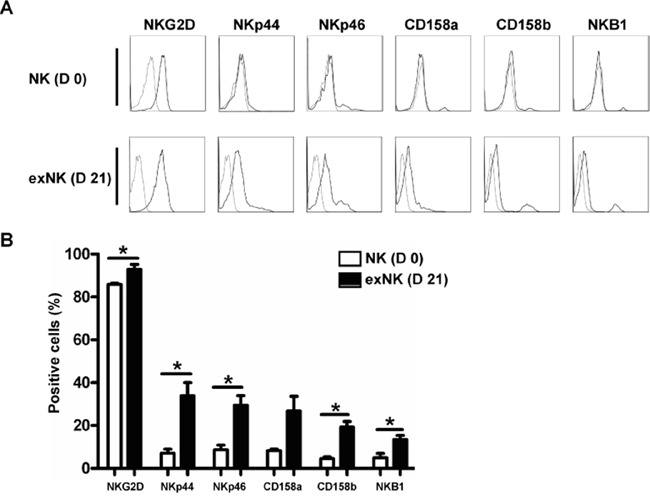
NK cell Phenotyping on day 0 and day 21 NK cell expression of activating receptors (NKG2D, NKp44 and NKp46) and inhibitory receptors (CD158a, CD158b and NKB1) on resting NK cells (D 0) and expanded NK cells (exNK) on day 21 of culture (D 21) were monitored by flow cytometry. **A.** Histograms from one representative experiment depicted expression of activating and inhibitory receptors on the resting NK (CD3-CD56^+^) and exNK cells. **B.** Expression of the activating and inhibitory receptors on the resting NK and exNK cells are shown as mean ± SEM (n=3, *P<0.05, by Student's *t* test).

NK cell expression of the activating receptors NKp44, NKp46 and NKG2D, and the inhibiting receptors CD158a, CD158b and NKB1 before expansion (day 0) and after 21-day culture (day 21) were analyzed by flow cytometry (Figure 2A). As shown in Figure 2B, not only activating receptor (NKG2D, NKp44 and NKp46) but also inhibiting receptor (CD158b and NKB1) expression on exNK cells was significantly increased. CD158a expression was elevated on exNK cells as compared to resting NK cells (NK cells before culture), albeit not statistically significant (P=0.13) (Figure 2B), which could be due to big variations in expression level on exNK cells and the small sample size.

### Increased degranulation and cytolytic activity of the expanded NK cells

Surface translocation assay of CD107a was used to determine NK cell degranulation activity. In general, the level of NK cell degranulation activity represents their killing capacity. Therefore, CD107a translocation assay could also be used to determine NK cell cytotoxicity in the condition of intact perforin expression [42].

**Figure 3:**
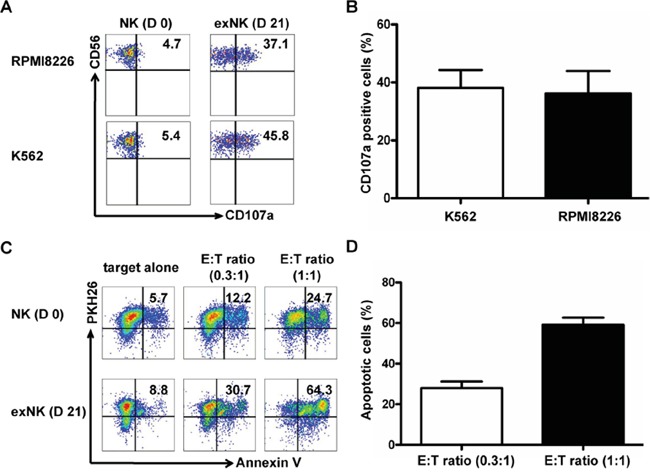
Degranulation and cytolytic activity of expanded NK cells CD107a translocation assay was used to evaluate degranulation activity of the resting NK (D 0) and expanded NK cells (exNK) on day 21 of culture (D 21) at effector:target (E:T) ratio of 1:1, and RPMI8226 and K562 cells were used as target cells. For evaluation of exNK cytolytic activity, RPMI8226 cells were stained by PKH26 prior to co-incubation with NK cells. PKH26 and Annexin V (AV) double positive cells were defined as apoptotic target cells of RPMI8226. **A.** The dot plots from one representative experiment illustrating CD107a surface expression on the resting NK and exNK cells after stimulation by the target cell lines, respectively. **B.** Results of degranulation capacity of the exNK cells from 3 independent experiments, K562 and RPMI8226 as target cells, respectively. **C.** Dots plots from one representative example depicted AV positive (apoptotic) RPMI8226 cells induced by the resting NK and exNK cells at Effector:Target (E:T) ratio of 0.3:1 and 1:1, respectively. **D.** ExNK cell cytolytic activity against RPMI8226 cells at E:T ratio of 0.3:1 and 1:1. The results are shown as mean ± SEM (n=3).

In these experiments, K562 cells, which are sensitive to NK cells-mediated killing, were used as a positive control, and CD3^−^CD56^+^CD107a^+^ cells were defined as degranulated NK cells. Our results showed that the exNK cells presented a strong degranulation capacity after cocultured with K562 and RPMI8226 as targets, respectively (Figure 3A and 3B).

In order to detect the killing capacity of resting NK cells and the exNK cells, the myeloma cells RPMI8226 stained with PKH26 were incubated with NK cells as described in the Methods. Apoptotic RPMI8226 cells were assessed by their Annexin-V binding with flow cytometry, and reported as the percentages of Annexin-V^+^PKH26^+^ cells in the total PKH26^+^ population. Figure 3C and 3D showed that exNK cells (day 21) markedly induced apoptosis of RPMI8226 cell (Annexin-V^+^ RPMI8226 cells) in a NK cell concentration-dependent manner.

### PD-L1 and PD-L2 mAbs blocking enhances the expanded NK cell cytotoxicity

**Figure 4:**
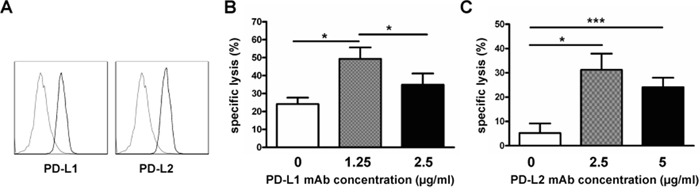
PD-L1/PD-L2 blocking enhanced expanded NK cell cytotoxicity Expanded NK cells on day 21 of culture (exNK) were collected and used as effector cells. RPMI8226 cells (target) were pretreated with or without PD-L1/2 blocking antibodies, and exNK cell killing activity was determined by the Colorimetric MTT assay. **A.** Representative histogram plots showing PD-L1 and PD-L2 expression on RPMI8226 cells (n=3). **B.** Comparison of exNK cell-mediated lysis of RPMI8226 cells treated by an anti-PD-L1 mAb at the indicated concentrations with E:T ratio of 1:1. Results are shown as mean ± SEM (n=3, *P<0.05, by Student's *t* test). **C.** ExNK cells-mediated lysis of RPMI8226 cells treated by an anti-PD-L2 mAb at the indicated concentrations with E:T ratio of 0.5:1. Values are shown as mean ± SEM (n=3, *P<0.05, ***P<0.001, by Student's *t* test).

Next, we evaluated the effect of blocking the PD1 ligands, PD-L1 and PD-L2. As shown in Figure 4A, PD-L1 and PD-L2 expression were detected on RPMI8226 cells by flow cytometry. Our results generated from three independent experiments showed that 58.5%±13.0% and 73.1%±7.5% of RPMI8226 expressed PD-L1 or PD-L2, respectively. To evaluate whether blocking PD-L1 and PD-L2 expression on RPMI8226 affects sensitivity of the myeloma cells to NK cell-mediated killing, RPMI8226 were first incubated with anti-PD-L1 or PD-L2 antibody before being added in the MTT assay as target cells. Interestingly, the lower concentration of the anti-PD-L1 antibody (1.25μg/ml) treatment resulted in more pronounced lysis of RPMI8226 mediated by the exNK cells than the control (without antibody) and higher antibody dose (2.5 μg/ml) treatment group, respectively (*P*<0.05 for the two comparisons made) (Figure 4B). Similarly, pre-blocking PD-L2 by anti-PD-L2 antibody at two concentrations (2.5μg/ml and 5.0μg/ml) tested significantly improved the exNK cell mediated lysis of RPMI8226 cells (31.2%±6.6% and 24.1%±3.9%, respectively) (Figure 4C). However, no dosage effects were observed in this setting of PD-L2 blocking (Figure 4C). These results indicated that blocking PD1/PD-L1 or PD-L2 interactions could be one effective way to improve NK cell therapy for MM or other tumors which are positive for PD-L1 or PD-L2 expression.

### PD1 expression on the expanded NK cells, and PD1 blocking enhances the expanded NK cell degranulation and cytolytic activity against myeloma cells

**Figure 5:**
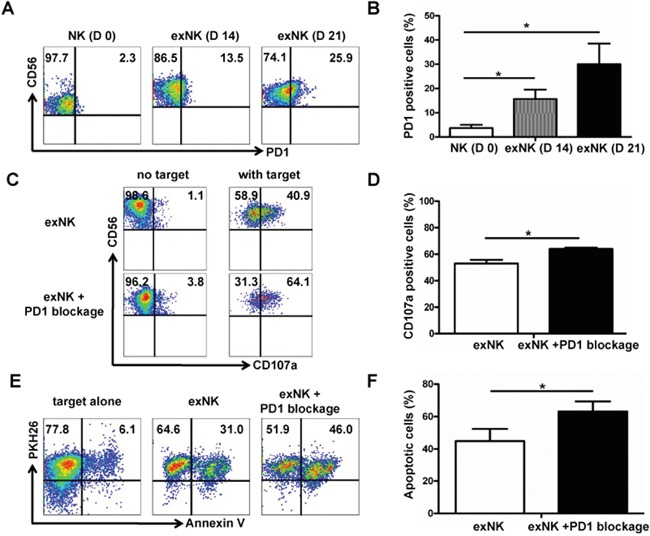
PD1 expression on expanded NK cells, and PD1 blocking enhanced exNK cell degranulation and cytolytic activity PD-1 expression on NK cells was tested on the time-points indicated using flow cytometry method. **A.** Dot plots from one representative experiment depicting PD1 expression on resting NK (D 0) and expanded NK (exNK) cells on day 14 (D 14) and 21 (D 21). **B.** Comparison of PD1 expression on the resting NK, exNK (D14) and exNK (D 21) cells. The degranulation (CD107a translocation assay) and killing activity of the exNK cells without PD1 blocking and with PD1 blocking by an anti-PD1 mAb (exNK+PD1 blockage cells) was compared, and MM cell line RPMI8226 was used as target cells in these assays by following the methods described in the Materials and Methods. **C.** Representative flow cytometry results showed CD107a positive (degranulated NK) cells: exNK cell spontaneous degranulation (no target) and degranulation triggered by the target cells (with target). **D.** Comparison of degranulated NK cells between exNK without PD1 blocking (exNK) and exNK-PD1 blockage cells. **E.** Dot plots from one representative example depicted spontaneous apoptosis and apoptosis of RPMI8226 induced by exNK and exNK-PD1 blockage, respectively. **F.** Comparison of apoptosis in the RPMI8226 cells induced by exNK and exNK-PD-1 blockage. Results from three independent experiments are shown as mean ± SEM (*P<0.5, by Student's *t* test).

PD1 expression on NK cells were tested on day 0, 14 and 21 using flow cytometry, respectively. Our results showed that PD1 was barely present on resting NK cells, but its expression was gradually induced along with the expansion process (Figure 5A). Approximately 26% of NK cells on day 21 of culture were positive for PD1 (Figure 5A). Percentages of PD1 positive NK cells on day 14 and 21 of culture were significantly higher than that on day 0, respectively (Figure 5B). The expression PD1 was numerically higher on day 21 than day 14, however the difference between these two groups was not significant (*P*>0.05) (Figure 5B).

**Figure 6:**
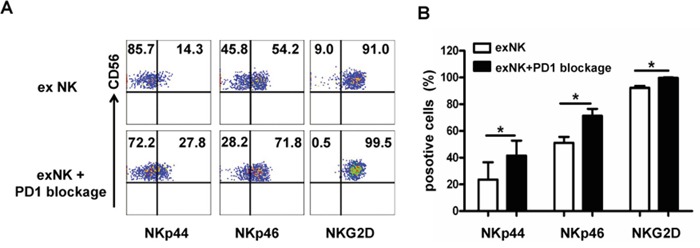
PD-1 blocking elevated activating receptor expression on expanded NK cells Expression of activating receptors (NKp44, Nkp46 and NKG2D) on the expanded NK cells without (exNK) and with PD1 blocking by an anti-PD1 antibody (exNK+PD1 blockage) in the *in vitro* expansion protocol was evaluated by flow cytometry method. **A.** Representative flow cytometry results showed NKp44, NKp46 and NKG2D expression on the exNK cells without and with PD1 blocking (exNK-PD1 blockage). **B.** Comparison of NKp44, NKp46 and NKG2D expression on the exNK cells without and with PD1 blocking (exNK-PD1 blockage). Results of three independent experiments are shown as mean ± SEM. (*P<0.5, by Student's *t* test).

To determine whether PD1 blocking in the NK cell expansion process could improve exNK cell cytolytic activity, we incorporated PD1 blocking by an antibody in our NK expansion protocol. As expected, our results clearly demonstrated that adding an anti-PD1 antibody into the expansion system markedly improved degranulation activity of NK cells upon incubation with RPMI 8226 as target cells (Figure 5C). Compared with the exNK cells without PD1 blocking in expansion system, our results showed that the exNK-PD1 blockage presented a significant higher percentage of degranulated (CD3^−^CD56^+^CD107a^+^) cells (64.0%±1.0% vs. 53.1%±2.7%, *P*<0.05) (Figure 5D). Consistently, exNK+PD1 blockage induced more pronounced apoptosis of RPMI8226 than the exNK without PD1 blocking (Figure 5E) and the difference between exNK and exNK+PD1 blockage groups was statistically significant (63.2%±6.2% vs. 44.9%±7.5%, *P*<0.05) (Figure 5F).

To elucidate the mechanisms underlying PD1 blockade-enhanced NK cell degranulation and cytolytic activity, we next examined the expression of several NK cell activation receptors using flow cytometry technique. Figure 6 showed that PD1 blocking markedly enhanced NKp44, NKp46 and NKG2D expression on the exNK cells (exNK+PD1 blockage) (Figure 6A). Moreover, the percentages of NKp44, NKp46 and NKG2D positive cells in the exNK+PD1 blockage group were significantly higher than those in the exNK group without the PD1 antibody in culture, respectively (Figure 6B)

### Adoptive transfer of the exNK, exNK+PD1 blockage and exNK cells plus PD-L2 blocking suppressed myeloma growth and improved survival of myeloma mice

**Figure 7:**
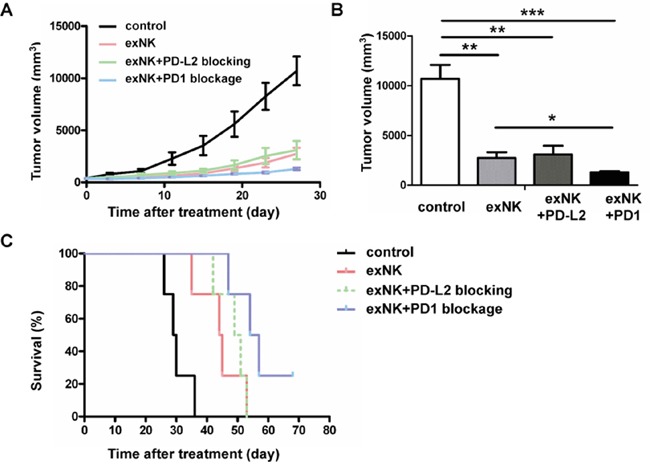
Expanded NK (exNK) cell transfusion suppressed tumor growth and prolonged survival of myeloma bearing mice ExNK and exNK+PD1 blockage cells, and normal saline (control) were transfused to the myeloma-bearing SCID mice by a tail vein. For exNK-PD-L2 blockage group, exNK cells were transfused by a tail vein combined with injection of anti-PD-L2 antibody intratumorly. **A.** The changes in tumor volumes among control and treatment groups at the observation time points indicated. **B.** Comparison of tumor volumes among the four different groups on the day 28 after treatments. The values are shown as mean ± SEM (*P<0.05, **P<0.01, ***P<0.001, by Student's *t* test). **C.** All the exNK, exNK-PD1 blockage and exNK-PD-L2 blockage treatments significantly prolonged survival of the myeloma bearing mice as compared with the control (*P < 0.05, **P < 0.01, by Long-rank test, respectively). Survival of the mice treated by exNK+PD1 blockage cells was significantly longer than the control (**P < 0.01, by Long-rank test) and exNK cells (*P < 0.05, by Long-rank test) respectively. There were four mice in each group.

To evaluate therapeutic effects of exNK cells, exNK+PD1 blockage and exNK+PD-L2 blocking with an anti-PD-L2 antibody, a MM mouse model was generated using SCID mice. As shown in Figure 4B and 4C, PD-L2 than PD-L1 blocking presented more efficient specific lysis-mediated by exNK cells, therefore the former approach was tested in this animal study. Our results demonstrated that between two and three weeks after subcutaneous inoculation of RPMI8226 cells, all sixteen mice developed tumors with a size approx. 300 mm^3^ and were randomly assigned into control, exNK, exNK-PD1 blockage and exNK+PD-L2 blocking groups, respectively. We found that tumor volumes in the control group grew more rapidly in a time-dependent manner than that in the exNK, exNK+PD-L2 blocking and exNK+PD1 blockage groups, respectively (Figure 7A). The exNK and exNK+PD1 blockage transfusion more rapidly sustained the tumor growth than the exNK+PD-L2 blocking. On week 2, 3 and 4 after treatment, all the mice from the three treatment groups showed markedly smaller tumor volumes than the control, respectively, and their differences between the groups compared the control were significant. To further highlight treatment effects of the NK therapies plus PD1/PD-L1 blocking, the changes in tumor volumes of the mice from four groups on day 28 after treatment (the date when the first mouse was died in the control group) were compared and presented in the Figure 7B. The results clearly demonstrated that tumor volumes of the mice treated by exNK, exNK+PD-L2 blocking and exNK+PD1 blockage were much smaller than the control, respectively (Figure 7B). The exNK+PD1 blockage group yielded much greater efficacy in suppression of tumor growth than exNK cells alone group (1297.0±118.1 vs. 2747.2±568.8, *P* < 0.05).

We further compared changes in survival among the control and treatment groups using Kaplan-Meier curve. Our results showed that the mean survival time of the mice treated by the control (saline) group, exNK, exNK+PD-L2 blocking and exNK+PD1 blockage were 30 days, 44 days, 49 days and 57 days, respectively. The last myeloma bearing mouse in control group was dead at day 36. The first mouse died in exNK, exNK+PD-L2 blockage and exNK+PD1 blockage groups was on days 35, 42 and 47, respectively. On day 53 after treatment, all the mice from the control, exNK, and exNK+PD-L2 blockage groups were died. As shown in Figure 7C, all three treatment regimens significantly prolonged survival of the myeloma mice when compared with the control. Moreover, exNK+PD1 blockage treatment significantly prolonged mice survival than the control (*P* < 0.01) and exNK alone (*P* < 0.05), and slightly prolonged the survival rate than the exNK+PD-L2 blockage (*P*<0.05). These data highlight that transfusion of exNK cells, exNK+PD1 blockage and exNK cells plus PD-L2 blocking on tumor cells are highly effective in suppressing tumor growth and improving survival of myeloma bearing mice, and exNK+PD1 blockage treatment appears to be most effective over the other two treatment approaches.

## DISCUSSION

NK cell-based immunotherapy emerges to be a promising therapeutic option for hematological malignancies and some solid tumors. Earlier trials of adoptive NK cell therapy employed methods including leukapheresis and magnetic bead-based isolation of NK cells with or without *in vitro* activation and/or expansion procedures [43]. The disadvantages of these methods are higher cost, more complicated procedures and requiring specialized processing equipments. CD16 engagement triggers potent NK cell activation and cytotoxicity against target cells [26, 44]. IL-2 is one of the key cytokines for induction of NK cell development, activation, proliferation and survival [45]. Compared to those protocols, the protocol presented in the current study using anti-CD16 antibody and IL-2 for *in vitro* expansion of NK cells is simpler. Moreover, PBMCs treated by our NK expansion protocol resulted in a large-scale of NK cell production with a good purity and potent cytolytic activity. More importantly, these expanded NK cells efficiently suppressed tumor progression and prolonged survival of the myeloma mice.

At present, optimal dosage of NK cells for therapy has not been determined. However, previous reports showed that there were no dose dependent side effects, and 1×10^8^-10^9^/kg of NK cells has been suggested as a relevant dose for future application [46, 47]. Based on this evidence, our protocol described herein could provide enough NK cells for NK cell adoptive treatment.

Adoptive transfusion of both autologous and allogeneic NK cells have been tested in clinic, and shown to be well tolerated in cancer patients [48–50]. NK cells express various activating receptors such as FcγRIIIA, activating forms of KIR (KIR2DS, KIR3DS), 2B4, NKG2D, NKp30, NKp44, and NKp46, and inhibitory receptors such as CD158a, CD158b, NKB1 (CD158e1) and NKG2A [51–53]. It is well known that lysis of target cells by NK cells is determined by the balance between the activation and inactivation signaling. Engagements of KIRs with their cognate ligand MHC I molecules expressing on cancer cells may result in inactivation of NK cells. One potential problem for using autologous NK cell therapy is that they can still recognize self-MHC I on tumor cells, which consequently hamper NK cell-mediated cytotoxicity toward tumor cells [17, 54, 55]. Our results showed that the expression of NK cell activating receptors (NKG2D, NKp44 and NKp46) analyzed were significantly up-regulated on the expanded NK cells. However, the expression of inhibitory receptors (CD158b and NKB1) tested were also significantly increased, which may negatively affect NK cell activation and killing activity. To overcome such matched barrier or upregulated expression of inhibitory receptors induced by activation, blocking KIR/MHC-I engagement using anti-KIR antibodies to create KIR/MHC-I miss-match or “allo-NK activity” environment could be one promising approach for enhancing NK cell-mediated killing of tumor cells and efficacy of NK cell adoptive therapy [56, 57]. Another way to induce allo-NK activity is to apply allogeneic haploidentical NK cells for therapy. Several studies have shown that infusion of haploidentical NK cells to exploit KIR/MHC alloreactivity in adults and children is safe and results in promising outcome in treatment of relapsed/refractory hematological malignancies especially acute myeloid leukemia (AML) and some solid tumors [15, 58]. However, using these approaches expensive equipments and supporting materials are required, which limits application of NK cell therapy in regular hospitals. In comparison with this methodology, our protocol has advantage over these.

The antibodies against PD1/PD-L1 have been tested in various solid tumors such as lung cancer, melanoma, kidney cancer and/ or hematological malignancies such as lymphoma. Those trials have shown great success in the treatment of those tumors [59–64]. PD1 expression on T/NK cells and myeloma cells have been previously reported, respectively [40, 65]. Our results showed a high level of PD-L1/L2 expression on myeloma cell line RPMI 8226, and PD-L1 and -L2 blocking on myeloma cells by the relevant blocking antibodies significantly improved the expanded NK cell cytotoxicity against myeloma cells *in vitro*. Moreover, PD-L2 blocking on RPMI8226 cells significantly improved the expanded NK cell-mediated suppression of tumor growth and prolonged the survival of myeloma mice, suggesting that adoptive NK cell therapy combined with *in vitro* or *in vivo* PD1/PD-ligand blocking may have a great clinical potential for cancers.

Our preliminary data has shown that PD1 expression on T cells in the bone marrow is significantly associated with tumor mass and survival parameters. We speculated that *in vitro* expansion procedures might amplify PD1 expression on NK cells. In line with this hypothesis, our results showed that PD1 expression on NK cells was found to be gradually induced during the process of *in vitro* NK cell expansion by using our NK cell expansion protocol. However, when additional PD1 blocking anti-PD1 antibody was added in the NK cell expansion system, the NK cells degranulation and killing activity against myeloma cells were significantly up-regulated. Moreover, these NK cells (exNK-PD1 blockage) significantly prolonged the survival of myeloma mice as compared to the mice treated with the expanded NK cells without PD1 antibody blocking. In line with these PD1 blocking associated-improvement in NK cell *in vitro* and *in vivo* activity, NKp44, NKp46 and NKG2D were significantly enhanced, which may at least in part contribute to the PD1 blocking associated improvement of anti-myeloma activity of exNK cells. Additionally, anti-PD1 antibody may increase migration of NK cells toward MM targets and enhances immune complex formation between NK cells and PD-L1–bearing tumor cells [40], which may improve treatment efficacy of NK cells *in vivo*.

One recent phase I study in MM demonstrated that PD1 blocking by anti-PD1 antibody did not show significant treatment benefit [66]. This highlights that PD1 blocking based-immunotherapies need to be further improved. The *in vitro* blockade of PD1 expression on *in vitro* expanded NK cells or adoptive transfer of NK cells combined with anti-PD1 antibody treatment may provide more beneficial therapeutic effects. However, to address this issue, clinical trials need to be carried out.

Genetic engineering of T cells to express chimeric antigen receptor (CAR) redirecting and amplifying their antitumor specificity and efficiency have been proven in clinical setting of B cell malignancies [67, 68]. Principally, engineering of NK cells to express CAR should improve their specificity against tumors. In view of the shorter lifespan and potent cytolytic function of mature NK cells, CAR-NK may result in efficient therapeutic effects with lesser severe cytokine storm, one fatal inflammatory condition associated with CAR-T therapy. Our NK cell expansion protocol combined with PD1 blockade may provide an efficient system for the generation of CAR-NK cells for adoptive therapy against such malignancies.

In summary, our present study has demonstrated an efficient and simple method to expand NK cells *in vitro* from peripheral blood mononuclear cells for NK cell adoptive therapies. The expanded NK cells markedly suppressed myeloma mass and prolonged the survival of myeloma mice. Moreover, Adding anti-PD1 antibody into our NK cell expansion system significantly improved NK cell cytotoxicity toward myeloma cells and NK cell transfusion-associated treatment efficacy in terms of suppression of tumor growth and a prolonged survival of myeloma mice. The present findings indicate that our NK cell expansion protocol combined with PD1 blockade may have a great clinical potential for cancer immunotherapies, and that the protocol provides a simple and efficient system for the therapeutical production of NK cells.

## MATERIALS AND METHODS

### Reagents

GMP grade CellGro® DC medium was purchased from CellGenix (Freiburg, Germany). Fetal bovine serum (FBS) was from ExCell Biology (Shanghai, China), while penicillin, streptomycin, and glutamine were all from Gibco (Waltham, MA, USA). Interleukin-2 (IL-2) was from Peprotech (Rocky Hill, NJ, USA).

Fluorescent labeled anti-human monoclonal antibodies (mAb) used for flow cytometric analysis were CD3-PerCP, CD16-FITC, CD56-PE, CD56-FITC, NKp44-PE, NKp46-PE, NKG2D-PE, CD158a-FITC, CD158b-FITC, NKB1-FITC, CD107a-FITC and relevant isotype control antibodies. The first five antibodies were purchased from Miltenyi Biotec (Bergisch-Gladbach, Germany), and the others from BD Pharmingen (San Diego, CA). LEAF^TM^ Purified anti-human CD16 antibody, purified anti-human CD274 (PD-L1), anti-human CD273 (PD-L2) and anti-human CD279 (PD1) antibodies were all purchased from Biolegend (San Diego, CA).

### Cancer cell lines

Myeloma cell line RPMI8226 and leukemia cell line K562 were both from ATCC (Manassas, VA, USA). The cells were cultured in RPMI1640 medium (Hyclone; Logan, Utah, USA) with 10% FBS and 100 U/ml penicillin-streptomycin as the target cells for testing the function of NK cells.

### NK cell expansion system

Heparinized human peripheral blood collected from volunteers was used as the source of NK cells. Briefly, PBMCs were isolated using Ficol (Tianjin HY Bioscience Co. LTD). After three washes, the cells were re-suspended with complete medium, CellGro® DC medium containing 10% FBS, 100 U/ml penicillin-streptomycin and 2 mM glutamine, then plated onto the six-well dishes at the concentration of 1×10^6^/well. The PBMCs were stimulated with anti-CD16 mAb, and IL-2 was added to the medium throughout the whole culture period with a concentration of 500U/ml. Medium was refreshed every other day. The cultured cells were transferred to a T25 flask or a cell culture bag (Takara, Japan) on day 9 depending on the volume of cell culture. At day 14 and 21, the cultured cells were collected and analyzed for NK cell purity, number and function.

### NK cell phenotyping

NK cell phenotype and expression of activating and inhibitory receptors were analyzed by flow cytometry. In short, PBMCs before culture (day 0) and after culture for 2-3 weeks were stained with the above fluorescence labeled antibodies for 30 mins at 4^°^C. The cells were washed in phosphate buffer saline (PBS), and then resuspended in 1% PFA-PBS buffer. Anti-CD279 mAb-PE was used to examine the PD1 expression on NK cells following the method described above. To monitor PD-L1 and PD-L2 expression on RPMI8226 cells, the cells were incubated with the corresponding mouse anti-human primary antibodies (anti-PD-L1 and anti-PD-L2 mAb) for 30 mins at 4^°^C. After two washes, the cells were further incubated with the secondary antibody (PE-conjugated donkey anti-mouse IgG) for another 15 mins at room temperature (RT). Afterwards, the cells were washed, resuspended in 1% PFA-PBS buffer, and analyzed by a LSR Fortessa flow cytometer (Becton Dickinson; Franklin Lakes).

### Degranulation assay

CD107a translocation assay was applied to detect perforin/granzymes containing granule degranulation activity of NK cells, as previously described elsewhere [69]. Briefly, 3×10^5^ PBMCs at day 21 of culture were mixed with 3×10^5^ K562 cells or RPMI8226 cells, and incubated at 37oC in 5% CO_2_. After 2 hour-incubation, the cells were spinned down, and stained with anti-human CD3-PerCP, CD56-PE, and CD107a-FITC mAbs in PBS with 2% FBS for 30 mins at 4^°^C. After two washes, the cells were resuspended in 1% PFA-PBS buffer and analyzed by BD LSR Fortessa flow cytometer.

### NK cell cytotoxicity assays

Apoptotic cell detection assay: Annexin-V-FlUOS (Roche, Manheim, Germany) was used to detect apoptotic cells induced by NK cells. Target cells RPMI8226 and K562 were first stained with PKH26 dye (Sigma, St. Louis, USA) according to the protocol provided by the manufacture. Briefly, 2×10^7^ target cells were collected, washed, and resuspended in 1ml Diluent C Solution containing 4μl PKH26 dye. The cells were stained for 5 min at room temperature in dark. Afterwards, the reaction was stopped by adding 2 ml human serum. After further incubation for 1 min, the cells were washed with the complete RPMI1640 medium twice and resuspended in the same medium. PKH26 stained RPMI8226 cells and K562 cells were co-cultured with NK cells (effectors) respectively at various Effector (E): Target (T) ratios for 4 h at 37^°^C with 5% CO_2_. Afterwards, the co-cultured cells were harvested, washed with PBS, and then resuspended in 100 μl Annexin-V-FLUOS labeling solution containing 2μl Annexin-V-FLUOS. After 15 mins incubation at RT, additional 400 μl buffer was added in each staining tubes. The percentage of specific lysis rate (PKH26^+^Annexin-V^+^) of RPMI8226 cells or K562 cells were monitored by a BD LSR Fortessa cytometer.

Colorimetric MTT assay was used to examine whether PD-L1 and PD-L2 mAb blockade could increase NK cell-mediated apoptosis of RPMI8226 cells. In the assay, the target RPMI8226 cells were first incubated with anti-PD-L1 mAb or PD-L2 mAb at the concentrations indicated for two hours at 37^°^C with 5% CO_2_. NK cells (1 × 10^5^) in 100 μl RPMI1640 culture medium were seeded into a 96-well microplate. PD-L1/PD-L2 mAb-pretreated or untreated RPMI8226 cells (control) in 100 ul RPMI1640 culture medium were then added to the wells at the E:T ratio of 1:1 or 0.5:1 in triplicates. The cells were gently mixed and incubated overnight at 37_o_C with 5% CO_2_. Afterwards, 20 μl of 5 mg/ml MTT solution was added into each well and cultured for another 4 hours. After this time, the plates were centrifuged, the supernatants were removed, and 150 μl of Dimethyl sulfoxide (DMSO) was added to each well. The microplates were shaken for 10 min, and the optical densities (OD) of dissolved formazan were then read at 570 nm using a microplate reader (Multiskan Mk3, Thermo). The percentage of specific lysis was calculated using the formula: [1-(Mean OD of co-cultured cells-Mean OD of Effector cells)/Mean OD of control cells] × 100%.

### NK cell expansion in the presence of anti-PD1 antibody

NK cells were expanded by our defined protocol, and anti-PD1 mAb was added to the expansion medium on day 7 of culture at the concentration of 2.5 μg/ml. The expanded NK cells (exNK) and PD1 blocked NK cells (exNK+PD1 blockage) were collected on day 21 of culture for phenotypic analysis, degranulation and cytotoxicity assays.

### *In vivo* experiments

The animal study was approved by the Animal Ethics Research Committee of the Second Hospital of Shandong University. SCID mice (female, 6-8 weeks old) were purchased from HFK Bioscience Co. (Beijing, China), and were housed in sterile laminar flow animal facilities. Human myeloma cell line RPMI8226 cells at logarithmic growth phase were collected and suspended in normal saline (NS) at 1×10^8^ cells/ml. Each mouse was injected subcutaneously near the right foreleg with 100 μl of the cell suspension. Treatment was initiated when tumor volume reached to approx. 300 mm^3^. Thereafter, the myeloma-implanted mice were randomly divided into four treatment groups: normal saline (NS, control), exNK cells, exNK+PD1 blockage, and exNK+PD-L2 blockage. The mice received NK cells (2×10^7^ cells/mouse) via tail vein injection, which was repeated after two days. In the arm of exNK+PD-L2 blockage, PD-L2 mAb was injected intratumorly (IT) at the dose of 20 μg/mouse one day before exNK transfusion. IL-2 (1×10^4^U/mouse) was intraperitoneally (i.p.) injected for two consecutive days after administration of the exNK cells or exNK+PD1 blockage. For a humanity reason, the mouse was euthanized by cervical dislocation when the mouse reached endpoint of our observation, which was defined as when a mouse was unable to creep for taking food and/or water. Tumor size and body weight were monitored every 3-4 days up to the day when the first mouse in control group reached the endpoint. Tumor volume was calculated by the formula: long diameter × (short diameter)^2^×π/6 [70]. At the end of the observation when the last mouse from one of the three (exNK, exNK-PD-L2 and exNK-PD1 blockage) treatment groups has reached endpoint (died), survival of the mice among control and treatment groups were compared using Kaplan–Meier survival curves.

### Statistical analysis

Student's *t* test and two-way ANOVA was used to compare differences between the groups. Long-rank test was carried out to analyze Kaplan-Meier survival curves. All statistical tests were performed with GraphPad Prism 5 (GraphPad Software Inc, San Diego, CA, USA). A difference was considered to be statistically significant when the two-sided *P* value was less than 0.05.
